# *Odorant binding protein 69a* connects social interaction to modulation of social responsiveness in *Drosophila*

**DOI:** 10.1371/journal.pgen.1007328

**Published:** 2018-04-09

**Authors:** Assa Bentzur, Anat Shmueli, Liora Omesi, Julia Ryvkin, Jon-Michael Knapp, Moshe Parnas, Fred P. Davis, Galit Shohat-Ophir

**Affiliations:** 1 The Mina & Everard Goodman Faculty of Life Sciences and Leslie and Susan Gonda Multidisciplinary Brain Research Center, Bar-Ilan University, Ramat-Gan, Israel; 2 HHMI Janelia Research Campus, Ashburn, VA, United States of America; 3 Department of Physiology and Pharmacology Sackler Faculty of Medicine, Tel-Aviv University, Tel-Aviv, Israel; 4 Sagol School of Neuroscience, Tel-Aviv University, Tel-Aviv, Israel; Washington University in Saint Louis School of Medicine, UNITED STATES

## Abstract

Living in a social environment requires the ability to respond to specific social stimuli and to incorporate information obtained from prior interactions into future ones. One of the mechanisms that facilitates social interaction is pheromone-based communication. In *Drosophila melanogaster*, the male-specific pheromone cis-vaccenyl acetate (cVA) elicits different responses in male and female flies, and functions to modulate behavior in a context and experience-dependent manner. Although it is the most studied pheromone in flies, the mechanisms that determine the complexity of the response, its intensity and final output with respect to social context, sex and prior interaction, are still not well understood. Here we explored the functional link between social interaction and pheromone-based communication and discovered an odorant binding protein that links social interaction to sex specific changes in cVA related responses. *Odorant binding protein 69a* (*Obp69a*) is expressed in auxiliary cells and secreted into the olfactory sensilla. Its expression is inversely regulated in male and female flies by social interactions: cVA exposure reduces its levels in male flies and increases its levels in female flies. Increasing or decreasing Obp69a levels by genetic means establishes a functional link between Obp69a levels and the extent of male aggression and female receptivity. We show that activation of cVA-sensing neurons is sufficeint to regulate Obp69a levels in the absence of cVA, and requires active neurotransmission between the sensory neuron to the second order olfactory neuron. The cross-talk between sensory neurons and non-neuronal auxiliary cells at the olfactory sensilla, represents an additional component in the machinery that promotes behavioral plasticity to the same sensory stimuli in male and female flies.

## Author summary

In this work, we used *Drosophila melanogaster* as a model organism to explore a basic question in neuroscience: why do different individuals experience the same sensory stimuli, such as smell differently, and moreover, why does one individual experience identical stimuli differently on different occasions? Focusing on sex specific behaviors in fruit flies, we identified *odorant binding protein 69a* (*Obp69a*) as a new player in the machinery that promotes behavioral plasticity to the same sensory stimuli in male and female flies.

## Introduction

A fundamental question in neuroscience is how do animals integrate sensory information, together with context, internal state and prior social interaction, into an appropriate behavioral response [1]. The manner by which prior social interaction affects behavioral responses is a well-described phenomenon associated with changes in gene expression [2, 3]. Causal links between past interactions, regulation of specific genes, and modulation of behavior, can be functionally dissected in model organisms that allow for genetic manipulation of genes and neuronal function, such as *Drosophila melanogaster*. We and others have previously demonstrated mechanisms in fruit flies by which social interaction shapes the expression of certain genes, leading in turn to long-lasting changes in behavior and physiology [4–6].

Social interaction is mediated by different mechanisms, one of which is pheromone communication, involving chemical cues that are emitted by one individual and perceived by another individual, predominantly of the same species (for review see [7]). In flies, sensory perception of pheromonal cues is mediated by olfactory and gustatory sensory neurons found within hair-like structures called sensilla. Odorant molecules are dissolved in the aqueous environment of the sensilla, where they bind to receptors located on dendrites of sensory neurons. This stimulates the neuron, which delivers sensory signal to the central nervous system (for review [7–13]).

Cis-vaccenyl acetate (cVA) is a male-specific *Drosophila* pheromone that was originally identified as an aggregation pheromone [14]. cVA elicits dimorphic responses in male and female flies, inducing aggression in the former and promoting sexual receptivity in the latter [15–20]. Currently, different innate responses to cVA exhibited by male and female flies are best explained by a wiring difference in the brain, whereby the third order sensory neurons project to distinct target neurons within the lateral horn [21].

In addition to innate responses, there are several examples where exposure to cVA induces different behavioral responses that depend on the context in which it is presented, and prior social encounters with other flies [22, 23]. For example, long-term exposure to cVA when male flies interact in a group reduces cVA-dependent individual aggression [24, 25], while exposure to cVA that is present on mated females, plays a role in memory formation for unsuccessful courtship in males, resulting in courtship suppression in future encounters [26–28]. This exemplifies the contextual component of the response to a single stimulus, the mechanisms by which it is achieved are still largely unknown.

cVA is sensed by Or67d and Or65a receptors in sensory neurons [15, 16, 18, 29]. cVA sensing also requires a soluble protein in the olfactory sensillar lymph, the odorant binding protein LUSH, which facilitates its movement through the lymph and its binding to Or67d receptors [30, 31]. Lush belongs to a family of 52 fly odorant binding proteins (Obps), the function of which is poorly understood [32, 33]. Insect Obps are globular proteins secreted from auxiliary cells that are located adjacent to olfactory and gustatory sensory neurons, and are believed to participate in facilitating the transport of hydrophobic odorants within the soluble environment of the sensillar lymph, or in their degradation [32–36].

Two independent studies identified transcriptional regulation of Obps in response to social stimuli [6, 37], the functional implication of which is not known. In this study, we identified an Obp family member, *Odorant binding protein 69a* (*Obp69a*) as a new player in the machinery that modulates behavioral responses to cVA. We demonstrate that *Obp69a* exhibits sexually dimorphic expression in fruit flies and is regulated inversely in male and female flies in response to similar social cues via the activation of cVA sensing neurons. Downregulating and upregulating Obp69a levels modulate cVA related behavioral responses oppositely in male and female flies, suggesting a link between prior social interaction, Obp69a levels and modulation of social responsiveness in future interactions.

## Results

### *Obp69a* is oppositely regulated in male and female flies

**Fig 1:**
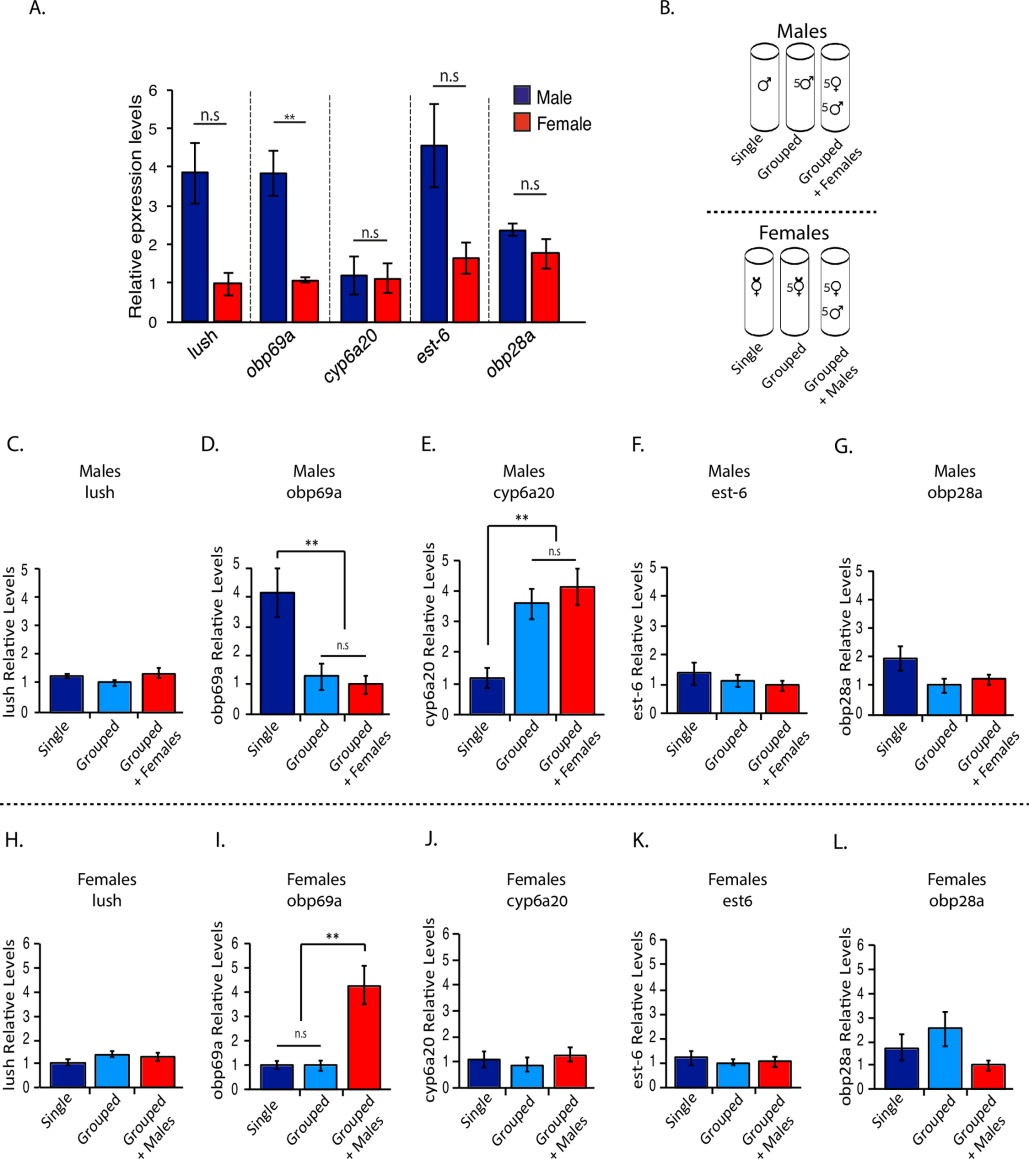
*Odorant binding protein 69a* (*Obp69a*) exhibits sexually dimorphic expression levels and is regulated inversely by social conditions in male and female flies. **A**. Male and female flies were housed separately in groups of 5 flies/vial for 3 days. Total RNA extracted from heads of grouped male and female flies was analyzed for mRNA levels of *lush*, *obp69a*, *cyp6a20*, *est-6 and obp28a* by RT-qPCR. Statistical significance was determined by Student’s T-test with Bonferroni correction for multiple hypothesis testing. Error bars signify SEM *P<0.001, n.s., not significant, n = 6 independent experiments with 10–15 fly heads/sample. **B.** Schematic illustration of social conditions set-up. WT males (upper panel) or females (lower panel) were housed individually, in groups of five same sex flies/vial or in groups of five male and five female flies for 3 days. **C-L.** Total RNA extracted from heads of male and female flies under single housing, same sex group and mixed sex group was analyzed for mRNA levels of *lush*, *obp69a*, *cyp6a20*, *est-6* and *obp28a* by RT-qPCR. Statistical significance was determined by one-way ANOVA with Tukey post-hoc analysis and Bonferroni correction for multiple hypothesis testing. (D) F(2, 6) = 9.4 **P<0.01. (E) F(2, 6) = 11.03 **P<0.01. (I) F(2,6) = 12.2 **P<0.01. n = 6 independent experiments of 15–20 fly heads/sample.

To further explore the previously identified connection between social conditions and odorant binding proteins in *Drosophila* [6, 37], we compared the expression levels of candidate genes between male and female flies, and in response to simple social conditions. Our analysis focused on genes with known functions within pheromone sensing sensilla (trichoid sensilla) such as *Lush* [30], *cyp6a20* [24] and *est-6* [38], and additional under-studied odorant binding proteins such as *Obp28a*, which was previously shown to be sensitive to courtship song [37], and *Obp69a*, which is known to be expressed in trichoid sensilla [32, 39] and is sensitive to environmental stimuli [40]. Pair-wise comparisons of candidate genes in total RNA extracted from heads of groups of five adult virgin male and five adult virgin female flies revealed an overall trend of higher expression levels in male flies (Fig 1A), however only *Obp69a* exhibited a significant difference when corrected for multiple comparison (Fig 1A *P*<0.01).

We next tested whether expression levels of the different candidate genes are sensitive to basic social conditions. Male and female flies were subjected to three simple social conditions over the course of three days post-eclosion. One cohort of flies (single) was subjected to social isolation from eclosion, a second cohort (grouped) was subjected to group housing in groups of five flies of the same sex, and a third cohort (grouped with females/males) was subjected to mixed-sex housing, i.e. five males and five females (see illustration in Fig 1B). Relative mRNA levels of each of the candidate genes was analyzed by RT-qPCR using total RNA extracted from intact heads (males, Fig 1C–1G and females, Fig 1H–1L). In male flies, a significant increase was documented in the relative levels of *Obp69a* in single-housed flies compared to grouped and grouped with females (Fig 1D *P*<0.01). No significant difference in relative *Obp69a* expression levels was observed between virgin male flies that were housed in groups and male flies that interacted and mated with female flies (*P*>0.05 Fig 1D). In agreement with Wang, et al. who described regulation of *cyp6a20* in response to social isolation [24], we detected a significant reduction in *cyp6a20* transcript levels in single housed male flies (Fig 1E *P*<0.01). In female flies, we observed a five-fold increase in *Obp69a* transcript levels in females that were housed with male flies, compared to single or grouped housed females (Fig 1I *P*<0.01). No significant difference in relative *Obp69a* levels was observed between the other two female cohorts (Fig 1I *P*>0.05). *Lush*, *Cyp6a20*, *est-6* and *obp28a* showed no significant expression difference across all conditions (Fig 1C, 1F–1H, 1J–1L P>0.05). This set of experiments suggests that in both male and female flies, exposure to male flies affects *Obp69a* expression, but in an opposite manner.

### *Obp69a* transcription is sensitive to male scents

**Fig 2:**
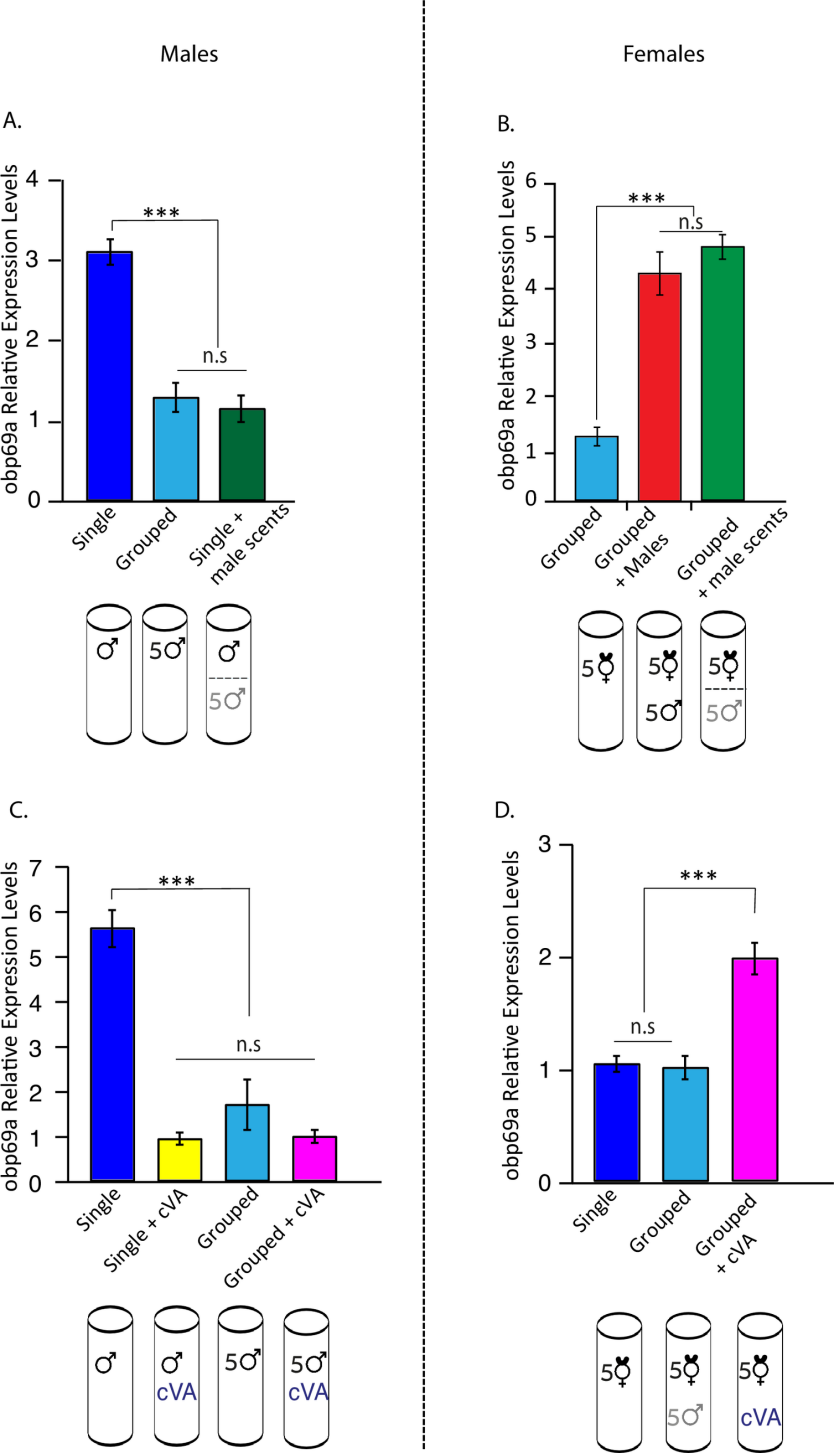
*Obp69a* transcription is dimorphically regulated in response to male scents, and exposure to the male pheromone cVA. Total RNA extracted from heads of male and female flies exposed to male scents (**A,B**) or to cVA (**C,D**) for three days was analyzed for *Obp69a* mRNA levels by RT-qPCR. Statistical significance was determined by one-way ANOVA with Tukey post-hoc analysis. Error bars signify SEM. ***P<0.001, (A) F(2, 6) = 28.5, (B) F(2, 6) = 44.1, (C) F(2, 6) = 24.3, (D) F(2, 6) = 28.3. n = 3 independent experiments of 15–20 fly heads/sample.

*Obp69a* is expressed in trichoid sensilla of the third antennal segment, the major olfactory sensory organ of the fly [32]. Therefore, we hypothesized that olfactory sensory signals, presumably pheromonal cues, might underlie the observed changes in *Obp69a* transcript levels. To test this, we asked whether exposure to male scents can induce *Obp69a* transcriptional change. Single housed male flies were exposed to other males through a mesh, restricting physical interaction but allowing odor, and possibly visual and auditory cues to pass, for the duration of three days. *Obp69a* expression levels were then measured and compared to single and grouped housed males (Fig 2A). Exposure to male scents was sufficient in reducing *Obp69a* expression levels, mimicking the effect of group housing (Fig 2A *P*<0.001). In female flies, analogous experiments revealed that *Obp69a* expression is also sensitive to male scents. Exposure to male cues via a mesh was sufficient in increasing relative *Obp69a* expression levels to similar levels as females that were grouped with males, suggesting the effects observed in females are not caused by mating, but rather by exposure to male signals that can pass through a mesh barrier, most probably olfactory cues (Fig 2B *P*<0.001). Control experiments were done to measure the levels of *lush* and *est-6* under the same experimental set up, revealing no significant regulation (S1A–S1D Fig, *P*>0.05). These results suggest that *Obp69a* transcription is regulated oppositely in male and female flies in response to a male signal, most likely a pheromone cue present in male scents.

### *Obp69a* transcript levels are sensitive to the male specific pheromone cVA

To identify the component in male scents that induces changes in *Obp69a* transcript levels, we took a candidate-based approach and tested whether exposure to cVA, a male specific pheromone, is sufficient in mimicking the transcriptional regulation of *Obp69a* in male and female flies following exposure to male scents. Single male flies were exposed to 10μg cVA over the course of three days, after which their relative *Obp69a* transcript levels were compared to those from single male flies that were exposed to the solvent, as a negative control, or to males housed in groups of five, as a positive control. A five-fold reduction in *Obp69a* expression levels was detected in single male flies exposed to cVA, compared to negative controls, similarly to that observed in the positive control (Fig 2C *P*<0.001). Exposing virgin females to 10μg cVA over the course of three days increased *Obp69a* transcript levels compared to females that were exposed to the solvent alone, mimicked the effect of exposure to male scents and housing with male flies (Fig 2D *P*<0.001). *lush* and *est-6* expression levels were also measured in response to cVA exposure, revealing no significant regulation in both cases (S1A–S1D Fig). So far, this data demonstrates that exposure to cVA is sufficient in affecting transcription of *Obp69a* oppositely in male and female flies, decreasing its expression in male flies and increasing its levels in female flies.

### *Obp69a* links prior social interaction to social responsiveness

**Fig 3:**
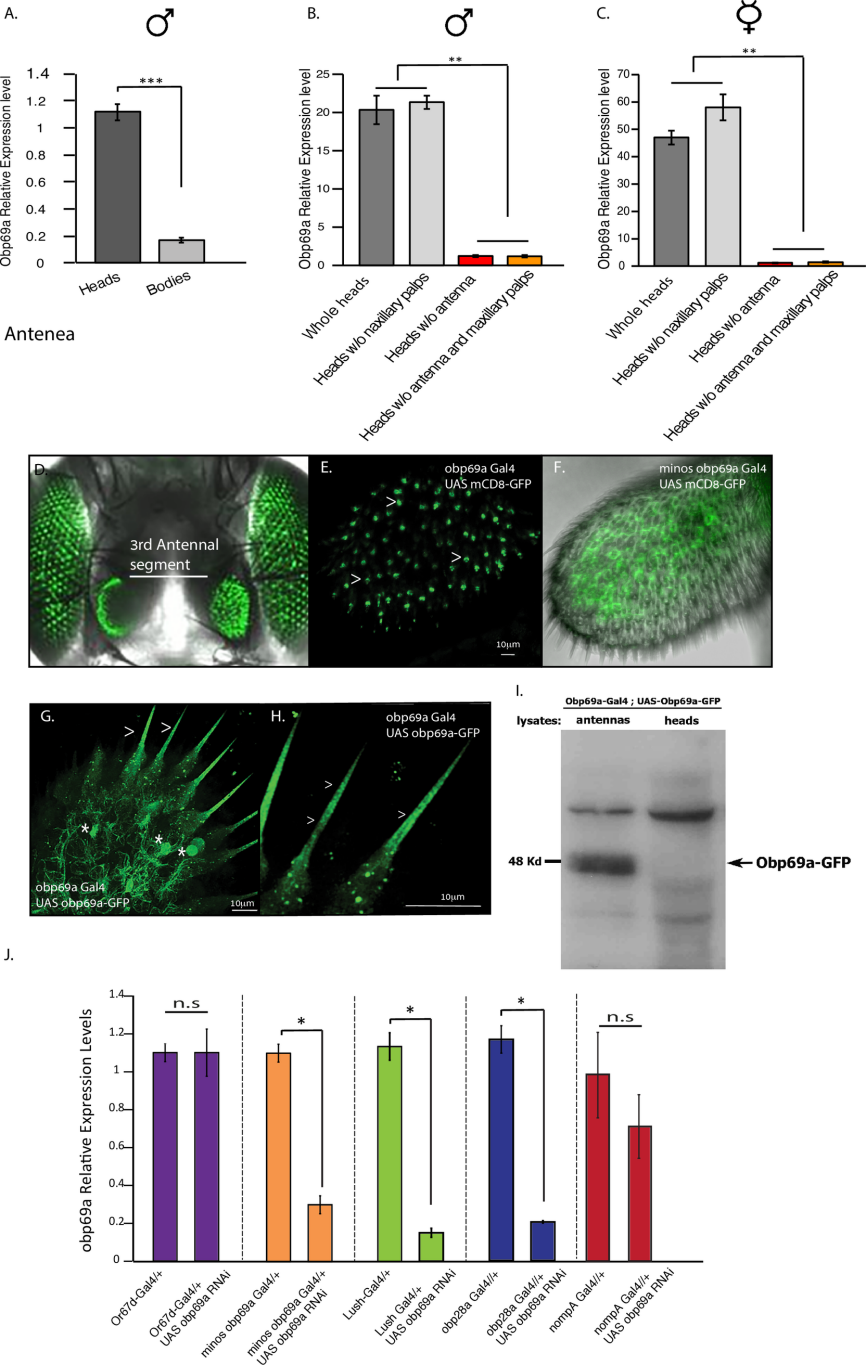
Obp69a is expressed in cells within the third antennal segment and is exported to the lymph. **A-C**. Relative Obp69a expression levels in male heads and bodies (**A**), and between heads without antennae or maxillary palps in males (**B**) and females (**C**). Statistical significance was determined using Student’s t-test (**A**) ***P<0.001, or One-way ANOVA with Tukey post-hoc analysis, Error bars signify SEM. (**B**)F(3,8) = 120, (**C**)F(3,8) = 124 **P<0.01. n = 3 independent experiments of 10–15 fly heads/sample. **D-H.** Confocal images of a membrane bound GFP (mcd8-GFP) in Obp69a expressing cells (*Obp69a-GAL4*), marking the 3^rd^ antennal segment. Note the GFP expression in the eyes is a marker of the *minos* element. (D). Arrowheads mark individual cells in antenna. E, F. Confocal images of a membrane-bound GFP (mcd8-GFP) in Obp69a expressing cells (using *Obp69a-GAL4* and *Minos Obp69a-GAL4* accordingly), marking the 3^rd^ antennal segment and presumably auxiliary cells. **G, H.** Confocal images of a transgenic Obp69a fused to GFP (*UAS-Obp69a-GFP*) expressed in Obp69a-expressing cells (Obp69a-GAL4). Asterisks mark Obp69a-GFP expression within the cells, Arrowheads mark exported Obp69a-GFP in the lymph. **I.** Western blot analysis of antennae and heads of *Obp69a-GAL4/+; UAS-Obp69a-GFP* flies using anti-GFP antibodies. **J**. RNAi to Obp69a was expressed in Or67d neurons, Lush, Obp69a, and Obp28a and nompA cells. *Obp69a* mRNA levels assessed by RT-qPCR. Statistical significance between relative mRNA levels in control and KD in each cell type was determined by Student’s T-test with Bonferroni correction for multiple hypothesis testing, Error bars signify SEM. *P<0.05, n.s., not-significant. n = 3 independent experiments of 10–15 fly heads/sample.

The observed sexual dimorphism in *Obp69a* gene regulation following exposure to cVA prompted us to ask whether *Obp69a* participates in the responses of male and female flies to social interactions that involve cVA sensing. To explore this direction, we first characterized Obp69a spatial expression pattern and the appropriate genetic tools for manipulating its levels. We compared Obp69a transcript levels that were isolated from heads and bodies and discovered that while it is expressed in both tissues, it is highly enriched in heads (Fig 3A *P*<0.001 and ModeEncode). Further dissections revealed that it is expressed in antenna, as removal of the antenna diminished its relative expression in male and female heads (compare whole heads to heads lacking antenna Fig 3B and 3C and also see *in situ* hybridization by Pikielny et al [39]). To explore this further, we used two GAL4 driver lines; a *Minos* transposable element inserted within the coding region of *Obp69a* (*Mi{ET1}Obp69a GAL4*), and a newly created *GAL4* line in which the coding sequence of *Obp69a* was swapped with GAL4 by homologous recombination. In line with previous work by Larter, et al., [32] membrane-bound GFP showed equivalent expression in the third antennal segment using both *Obp69aMi-GAL4* and *Obp69a-GAL4* drivers (Fig 3E and 3F). Expressing a newly created GFP-fused version of Obp69a resulted in fluorescent signal within cells and the sensory sensilla (Fig 3G and 3H), suggesting that Obp69a is produced in auxiliary cells and secreted to the sensillar lymph. Expression of *Obp69a-GFP* was also validated using Western-Blot analysis (Fig 3I).

To test our ability to modulate Obp69a expression in the relevant anatomical context, we expressed *Obp69a-RNAi* in different cell types, and assessed *Obp69a* transcript levels. Driving *Obp69a-RNAi* using *Mi{ET1}Obp69a* GAL4 (the *Minos* insertion does not impair *Obp69a* expression) resulted in more than five-fold reduction in *Obp69a* levels (Fig 3J *P*<0.05). A significant reduction of *Obp69a* expression was also observed in LUSH positive cells and in Obp28a positive cells (Using *Lush GAL4* and *Obp28a GAL4*, respectively, Fig 3J *P*<0.05), but not when using *Or67d GAL4* or *nompA GAL4* (expressed in sensory neurons and techogen type of auxiliary cells, respectively) (Fig 3J *P*>0.05). These results reinforce previous findings [32] showing that Obp69a is expressed in non-neuronal cells of the thormogen subtype in the antennae, and suggest that Obp69a is mutually expressed with LUSH in cVA sensing sensilla (which harbors Or67d receptor).

Having the tools to manipulate the expression of Obp69a specifically in Obp69a producing cells, we proceeded to test whether it plays a role in cVA related social interactions. cVA sensing is necessary for adequate sexual receptivity in female flies, and promotes aggressive interaction in male flies [29]. In addition, prolonged exposure to cVA, a normal outcome of male group housing, reduces aggressive behavior, while social isolation induces it [25].

**Fig 4:**
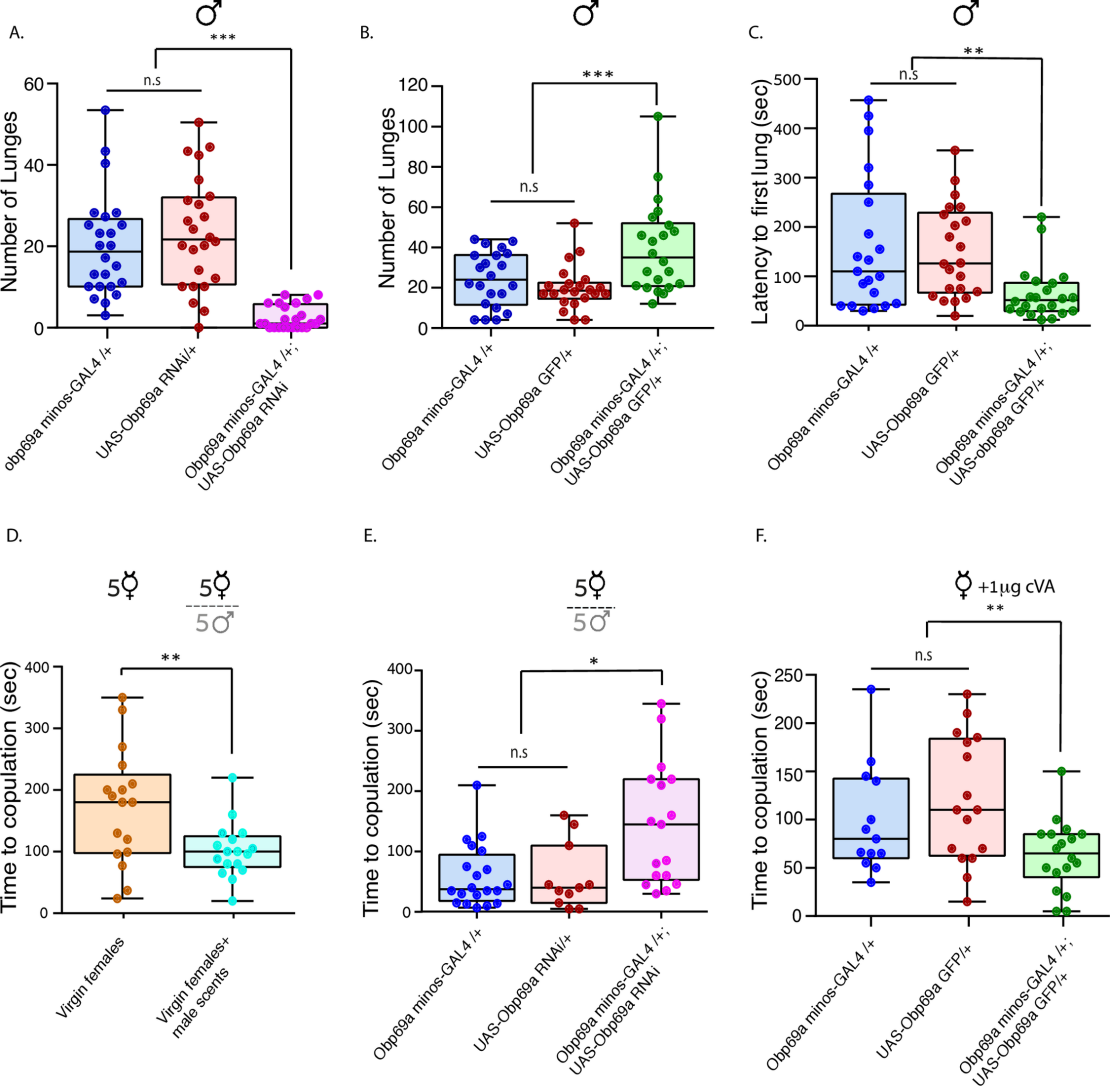
Obp69a links prior social interaction to modulation of social responsivity. **A**. Obp69a was knocked-down by RNAi in male flies and number of lunges was scored. Statistical significance was determined by one-way ANOVA with Tukey post-hoc analysis, F(2, 69) = 25.4 ***P<0.001, n = 24. **B, C**. Obp69a was over-expressed in male flies and the number of lunges was scored (**B**) or the time until the first lunge (latency) was measured. Statistical significance was determined by one-way ANOVA with Tukey post-hoc analysis. (B) F(2, 63) = 8.5, (C) F(2, 62) = 6.2 **P<0.01, ***P<0.001, n = 24 **D.** Female flies were exposed to male scents prior to the courtship assay, and the time to copulation with WT virgin male flies was measured. Statistical significance was determined by Students t-test. **P<0.01, n = 17. **E.** RNAi to Obp69a was expressed in females that were previously exposed to male scents, and the time to copulation with WT virgin males was measured. Statistical significance was determined by one-way ANOVA with Tukey post-hoc analysis, F(4, 42) = 3.55, *P<0.05, n = 18. **F.** Obp69a-GFP was expressed in female flies, which were exposed to 1μg cVA prior to mating with WT virgin males. The time to copulation was measured. Statistical significance was determined by one-way ANOVA with Tukey post-hoc analysis, F(2, 44) = 4.9, **P<0.01, n = 34, 42 and 31 for *Obp69aMi-GAL4*, *UAS-Obp69a-GFP* and *Obp69aMi*-*GAL4*; *UAS-Obp69a-GFP* respectively.

To determine whether the correlation between *Obp69a* transcript levels and the behaviors that are associated with the different social conditions reflects a cause-and-effect relationship, we used *Obp69a* KD or over-expression to reproduce the expression levels observed in group housing and social isolation, respectively. If Obp69a participates in modulating aggression along the social isolation-group housing axis, decreasing its levels in single housed male flies should reduce the extent of aggressive displays. Hence, we measured males’ aggression under naturalistic conditions that rely on the presence of endogenous cVA on rival male flies [16, 25]. Single male flies in which the levels of *Obp69a* were down-regulated by RNAi exhibited dramatic reduction in aggressive behavior, as measured by the number of lunges in 30 min, compared to genetic controls (Fig 4A *P*<0.001). Since grouped male flies rarely exhibit aggression, we chose to test whether over expressing Obp69a can enhance aggression in single male flies. Increasing Obp69a levels via expression of Obp69a-GFP significantly induced aggressive displays, as reflected by increased number of lunges (Fig 4B *P*<0.001) and shortened the latency to first aggressive display (Fig 4C *P*<0.01). Thus, increasing the levels of Obp69a can enhance aggressive behavior. This implies that changes in *Obp69a* levels, most likely in the antenna, can regulate the rate of aggressive displays, in which down-regulation decreases, and up-regulation increases aggressive behavior in single male flies.

To test the behavioral relevance of *Obp69a* transcriptional regulation in female flies, we first validated that exposure to male scents without mating promotes sexual receptivity. Virgin Wild-Type (WT) female flies were exposed to male scents over the course of three days via mesh, and their sexual receptivity was subsequently assessed in a courtship assay by measuring the time until copulation from the moment the male partner exhibited the first courtship display. A 50% increase in receptivity was observed in meshed females compared to controls (Fig 4D *P*<0.01), indicating that exposure to male scents, which upregulates *Obp69a* transcription in females, facilitates receptivity in female flies.

To determine whether the correlation between *Obp69a* transcript levels and cVA induced receptivity is causally linked, we reduced or increased Obp69a expression in female flies and measured receptivity towards mature male flies. Down-regulation of *Obp69a* by expressing *Obp69a-RNAi* resulted in no significant changes in sexual receptivity, compared to genetic controls (S2A Fig, *P*>0.05). However, when female flies were exposed to male scents shortly before introducing a male partner, a significant reduction in sexual receptivity was observed in the experimental group (Fig 4E *P*<0.05), suggesting that *Obp69a* is necessary for promoting receptivity in response to cVA exposure.

Increasing Obp69a levels by means of expressing *Obp69a-GFP* in the absence of previous exposure to cVA did not affect female receptivity (S2B Fig, *P*>0.05). However, short exposure to a sub-optimal concentration of cVA (1μg), significantly shortened the time required to reach successful copulation compared to genetic controls (Fig 4F *P*<0.01). Our results in female flies imply that Obp69a can alter the magnitude of the stimulating effect of cVA exposure on sexual receptivity.

### The activity of cVA sensing neurons is necessary and sufficient for *Obp69a* transcriptional regulation

**Fig 5:**
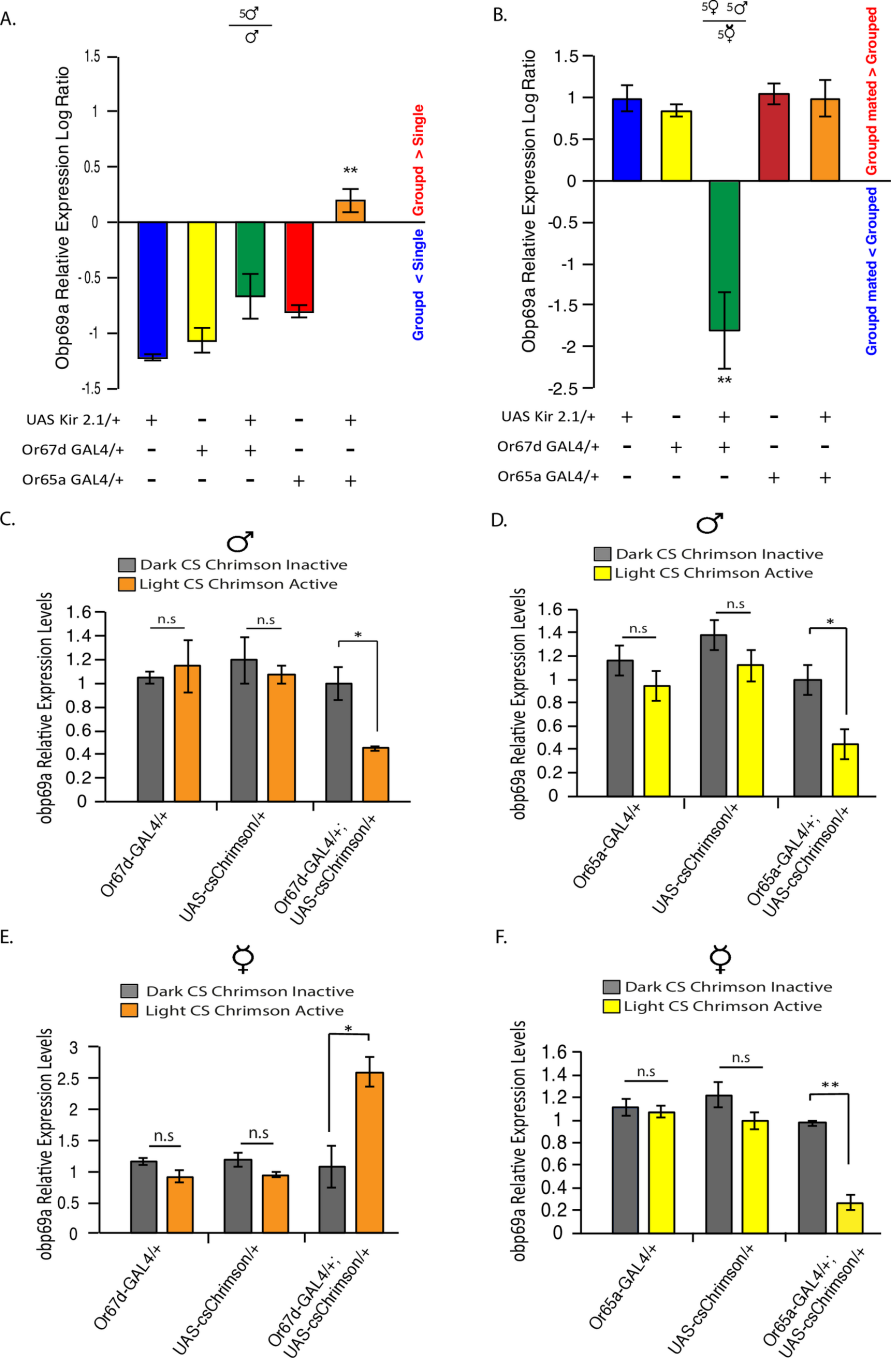
The activity of cVA sensing neurons is necessary and sufficient for regulating *Obp69a* transcription. **A, B.** Activity of Or65a and Or67d expressing neurons are necessary for Obp69a transcription changes in male and female flies. **A.** Relative *Obp69a* levels in single and Grouped housed male flies expressing *UAS-Kir2*.*1* in Or67d or Or65a neurons was analyzed using RT-qPCR. Log ratio represents the fold change in Obp69a relative levels of group divided by single housed. **B.** Relative *Obp69a* levels in female flies expressing *UAS-Kir2*.*1* in Or67d or Or65a neurons and subjected to group hosing or group housing with male flies was analyzed using RT-qPCR. Log ratio represents the fold change in Obp69a relative levels of grouped divided by grouped with males. Statistical significance was determined by One-way ANOVA with Tukey post-hoc analysis. Error bars signify SEM. (**A**) F(4,10) = 17.26, (**B**) F(4,10) = 15.5 **P<0.01, n = 3 independent experiments of 10–15 fly heads/sample. **C-F**, activation of cVA sensory neurons is sufficient in eliciting Obp69a transcriptional changes. Male (**C,D**) and female (**E,F**) flies expressing *CsChrimson* in Or67d (**C,E**) or Or65a (**D,F**) neurons (*Or67d-GAL4*, *UAS-Cs-Chrimson* and *Or65a-GAL4*, *UAS-Cs-Chrimson* respectively), and genetic controls were either subjected to spaced three 15-min long optogenetic activation sessions or were kept in the dark, after which *Obp69a* transcript levels were analyzed using RT-qPCR. Statistical significance was determined by Student’s T-test with Bonferroni correction for multiple hypothesis testing. Error bars signify SEM. *P<0.05, **P<0.01 (C,E,F) n = 4, (D) n = 5. n represents the number of independent experiment of 10–15 fly heads/sample.

cVA is sensed by two types of olfactory receptor neurons (ORNs): Or67d neurons and Or65a neurons. The former mediates acute responses to cVA, and the latter mediates chronic responses [15, 18, 25, 29, 41]. To identify which of the two neurons is relevant for cVA dependent regulation of Obp69a, we used the potassium-rectifying channel Kir2.1 to inhibit the activity of cVA sensing neurons in single and grouped housed male flies. Inhibition of the relevant sensory neuron is expected to diminish the difference in *Obp69a* levels between the two conditions. The regulation of *Obp69a* levels by single and grouped housing was maintained in male flies in which Or67d neurons were inhibited (Fig 5A). In contrast, inhibiting Or65a neurons diminished the difference in *Obp69a* levels between single and grouped flies (compare *Or65a GAL4/+; UAS Kir2*.*1/+* to the corresponding genetic controls, Fig 5A *P*<0.01). This result suggests that the activity of Or65a in male flies is necessary to reduce *Obp69a* levels in response to the presence of other males.

Performing similar experiments in female flies, analyzing the fold-difference between virgin female flies and female flies that were housed with male flies, revealed that inhibition of Or65a does not block the induction in *Obp69a* levels in response to interaction with male flies (Fig 5B). Surprisingly, inhibition of Or67d neurons in female flies did not only prevent the increase in *Obp69a* in female flies that interacted with male flies, but also led to a significant reduction in *Obp69a* levels (Fig 5B *P*<0.01). This could be explained by opposing effects of Or65a and Or67d neurons, whereby Or67d neurons are necessary for *Obp69a* induction in response to cVA, and Or65a neurons reduce its expression, an effect only revealed when Or67d neurons are inhibited.

To test whether activating cVA sensing neurons is sufficient to elicit changes in *Obp69a* transcript levels, we expressed the red-shifted channel rhodopsin CsChrimson [42] in Or67d or Or65a expressing neurons, and subjected flies to three 15 min long optogenetic activation sessions, after which relative *Obp69a* expression levels were analyzed. Notably, a 2-fold decrease in relative *Obp69a* mRNA levels was observed in naïve male flies following artificial activation of either Or67d or Or65a neurons, when compared to similar flies that were kept in the dark (no activations) (Fig 5C and 5D, *P*<0.05). *Obp69a* expression levels did not change in genetic controls subjected to similar conditions (Fig 5C and 5D, *P*>0.05). The changes in *Obp69a* expression in response to activation of Or67d or Or65a neurons was only evident in single male flies, as the activation protocol conducted on grouped housed male flies did not affect *Obp69a* transcript levels (S3A and S3B Fig, *P*>0.05), possibly as a result of chronic endogenous activation of these neurons during group housing [25]. Activating Or67d neurons in virgin female flies led to a significant increase in *Obp69a* expression levels compared to controls (Fig 5E *P*<0.05), while activation of Or65a neurons resulted in a three-fold decrease in *Obp69a* expression levels (Fig 5F *P*<0.01). The opposing effect of Or65a and Or67d neuronal activation on *Obp69a* transcription in female flies, together with the results from the inhibition experiments, suggests that in female flies, exposure to cVA and activation of Or67d neurons increases *Obp69a* levels, while activation of Or65a neurons decreases its levels. Altogether, the effects of neuronal activation on *Obp69a* levels imply a causal link between cVA exposure and regulation of *Obp69a* via the activity of cVA sensing neurons.

### Obp69a transcriptional regulation requires active neurotransmission of the information from the sensory neuron to the second order olfactory neuron

The regulation of *Obp69a* by cVA exposure and optogenetic activation, together with the fact that *Obp69a* is expressed in non-neuronal auxiliary cells prompted us to further explore possible mechanisms by which neuronal activation regulates *Obp69a* expression in auxiliary cells. There are two possible models that can account for the interplay between neuronal activation and transcriptional regulation in auxiliary cells: (a) a local direct interaction between the sensory neuron and auxiliary cells that converts neuronal activation to transcriptional changes within nearby auxiliary cells. (b) a non-direct mechanism in which sensory information is relayed to downstream neurons and eventually reaches auxiliary cells, perhaps via an afferent mechanism. To discriminate between the two models, we induced depolarization of cVA sensing neurons via optogenetics, and at the same time blocked synaptic vesicle release to downstream neurons using shibire^ts^. If the interplay between sensory neuron and auxiliary cell is based on an indirect afferent mechanism, depolarizing the sensory neurons while inhibiting synaptic vesicle release is expected to block information flow to auxiliary cells and produce no transcriptional change. Conversely, if the mechanism relies on direct interaction between the two, blocking synaptic vesicle release is not expected to suppress the depolarization effects, and thus *Obp69a* levels are expected to change.

**Fig 6:**
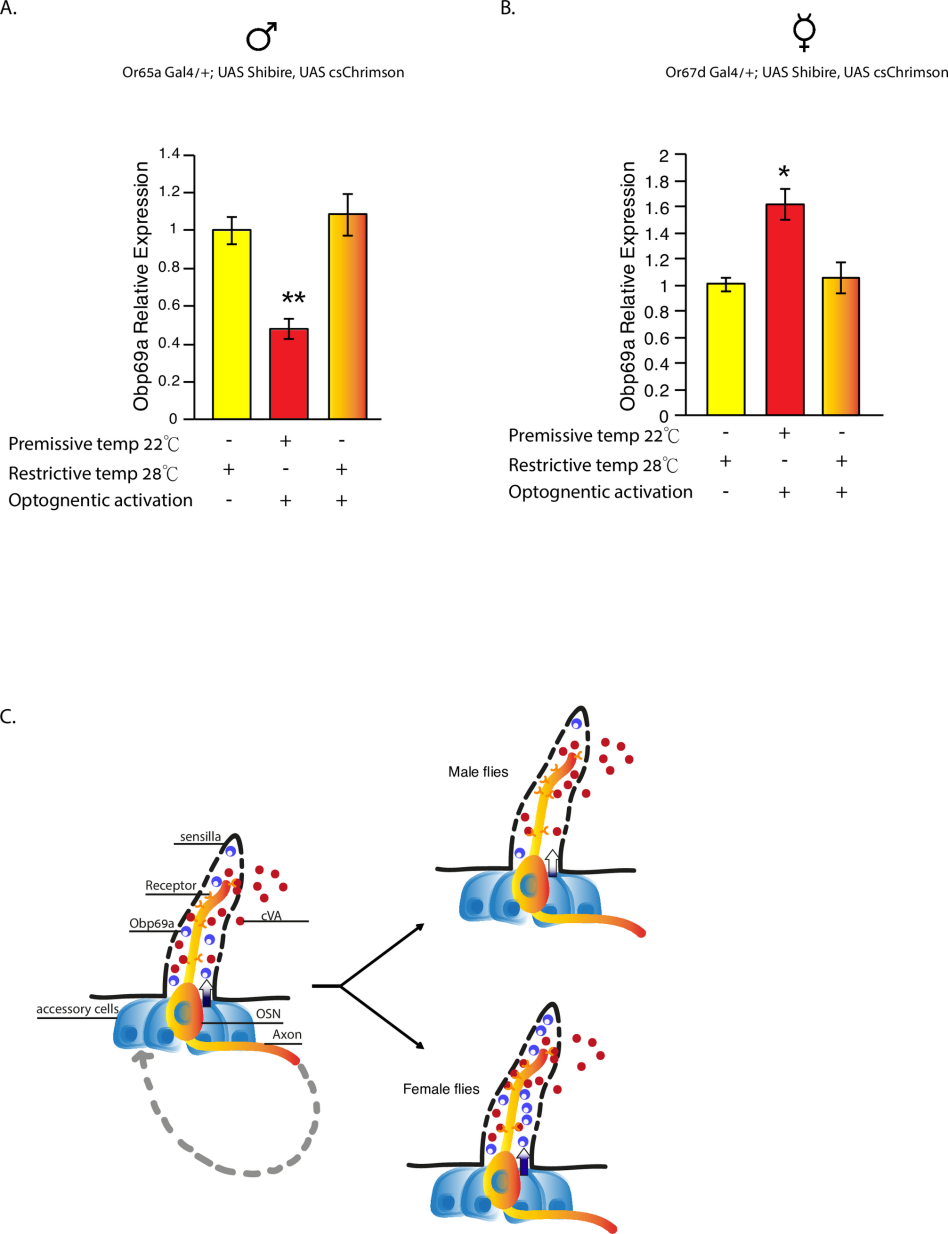
*Obp69a* transcriptional regulation requires active neurotransmission of the information from the sensory neuron to the second order olfactory neuron. (**A**) Male flies expressing *UAS-Cs-Chrimson* and *UAS-Shibire*^*ts*^ in Or65a neurons where subjected to three 15 minutes long optogenetic activations as a positive control, synaptic release blocking, or both. Obp69a relative expression was then measured using RT-qPCR. (**B**) Female flies expressing *UAS-Cs-Chrimson* and *UAS-Shibire*^*ts*^ in Or67d neurons where subjected to three 15 minutes long optogenetic activation as a positive control, synaptic release blocking, or both. Obp69a relative expression was then measured using RT-qPCR. Statistical significance was determined by One-way ANOVA with Tukey post-hoc analysis. Error bars signify SEM. (A) F(2,6) = 21.18, (B) F(2, 6) = 7.9, *P<0.05, **P<0.01 n = 3 independent experiments of 10–15 fly heads/sample. (**C**) Proposed model in which exposure to cVA regulates the production of Obp69a in accessory cells oppositely in male and female flies, via a mechanism that depends on relaying the information from the sensory neurons to the second order olfactory neurons in the brain, and eventually back to Obp69a producing cells.

To this end we expressed *UAS-csChrimson* and *UAS-Shibire*^*ts*^ in Or65a neurons and compared *Obp69a* levels in male flies that were subjected to three conditions: optogenetic activation (positive control), inhibition of synaptic vesicle release in the absence of activation (negative control), and combined activation and inhibition of synaptic vesicle release. While activation of Or65a neurons decreased *Obp69a* relative levels, the other two conditions resulted in no change in Obp69a levels (Fig 6A *P*<0.01). This result suggests that information transfer from Or65a neurons to the second order olfactory neurons is necessary for regulating *Obp69a* levels, supporting the indirect model. However, this does not preclude the existence of a local connection that depends on exocytosis from the neuron to the auxiliary cell, which would also be blocked by Shibire^ts^.

Similar activation and inhibition of synaptic vesicle release experiments performed on Or67d neurons in female flies indicated that the induction of *Obp69a* transcription in response to optogenetic activation is blocked by inhibition of synaptic vesicle release as well (Fig 6B *P*<0.05). Altogether, this set of experiments suggests that the dimorphic regulation of *Obp69a* in auxiliary cells requires active neurotransmission from the sensory neuron to the second order olfactory neuron, somehow conveying the information back to Obp69a producing cells.

## Discussion

The ability to incorporate experience obtained from prior interaction into a behavioral response is critical for survival and reproductive success. Here we used *Drosophila* to investigate mechanisms that link prior social interaction to modulation of sex specific pheromone communication and discovered *Obp69a* as novel player in the machinery that connects social interaction to modulation of sex-specific behaviors. *Obp69a* exhibits higher expression levels in male flies compared to females, a difference that can be explained by the slightly larger number of pheromone-sensing trichoid sensilla in male flies [43]. Regardless of this, *Obp69a* transcription is inversely regulated in males and females in response to cVA or to artificial activation of cVA sensing neurons in the absence of cVA, suggesting a causal link between cVA perception and *Obp69a* transcriptional regulation. In male flies, the activity of Or65a neurons is necessary to reduce Obp69a in response to cVA exposure. In female flies, Or67d and Or65a neurons have an opposite regulatory effect on Obp69a expression. This may be related to the different roles of cVA as a pheromonal cue under different mating states; serving as an attractant for virgin females but losing its attractive value after mating [41].

Until now, the dimorphic behavioral responses to cVA were thought to depend mostly on dimorphic wiring of the third order sensory neurons to distinct target neurons in male and female brains [21]. Our findings suggest an additional layer to this equation, showing that the soluble environment of the olfactory sensilla is different between male and female flies. The mechanism that converts activation of cVA sensing neurons into regulation of *Obp69a* within the auxiliary cells is not known. Nonetheless, our data imply that it depends on active neurotransmission from the sensory neurons to the second order olfactory neurons in the brain, and eventually back to Obp69a producing cells (see model in Fig 6C). Still, it is not clear whether the opposite regulation of *Obp69a* in female and male flies results from wiring differences that relay the information to auxiliary cells, or from inherent dimorphic transcriptional programs within Obp69a producing cells.

We used genetic manipulations of *Obp69a* to mimic the effects of social conditons on its expression levels and to explore the behavioral consequences of its modulation. Manipulating Obp69a expression to generate low levels, as in grouped housed flies, decreased aggressive displays in single housed male flies, while conversely, high levels of Obp69a facilitated aggression. The connection between Obp69a levels and aggressive behavior, together with its co-expression with LUSH, suggests that Obp69a plays a role in the machinery that generates aggressive behavior in the presence of cVA. This is consistent with previous work by Billeter, et al., which proposed the existence of a LUSH-independent cVA detection system [23]. These results propose that long exposure to cVA during group interaction which reduces *Obp69a* levels along with other physiological changes such as *Cyp6a20* up-regulation, may participate in reducing aggression, to promote aggregation and to allow mating. In female flies, down-regulation of Obp69a reduced receptivity, while over-expression boosted receptivity upon short exposure to suboptimal levels of cVA. This suggests, that regulating *Obp69a* levels can fine-tune the responsiveness of virgin female flies to the presence of male flies, promoting their receptivity.

The genetic approach used in this study, is limited in that it does not prove a direct causal link between the effect of social conditions on *Obp69a* levels, and subsequent modulation of behavioral response in future interactions. In other words, it is possible that the cVA dependent changes in *Obp69a* levels and the modulation of behavior in response to decreasing or increasing Obp69a levels, represent two independent processes. Nonetheless, considering the causal link between cVA sensing and Obp69a regulation, the causal relationships between Obp69a levels and the extent of male aggression and female receptivity in response to cVA, and the fact that these changes correspond to changes in behavior that happen naturally following exposure to cVA, we propose a model to combine the two parts: exposure to cVA during social interactions regulates *Obp69a*, which in turn participates in modulating cVA-dependent behavioral responses in future interactions, suggesting the existence of a feedback loop linking cVA and Obp69a. This may serve to integrate prior interactions in the form of cVA concentration, and presumably time of exposure, into sensitivity to the same pheromone on future encounters (see model in Fig 6C). The biochemical mechanism that shapes these responses still needs to be resolved, including whether Obp69a binds cVA directly, and whether it interacts with other players within the sensilla such as the receptor, LUSH and odorant degrading machinery.

The proposed modulatory function of Obp69a is not the first example in which Obps modulate behavioral response to a certain stiumilus. Previously, it was shown that Obp56h can modulate mating behavior [44]. Another odorant binding protein in *Drosophila*, Obp49a, was shown to act in sugar sensing sensilla to inhibit responses to sugar in the presence of bitter compounds [45]. This, along with the fact that the principles by which olfactory information is processed within the nervous system is conserved from fruit flies to mammals, suggests that the functional role of Obps may also be conserved. In vertebrates, the nasal mucus consists of abundant levels of odorant binding proteins, the function of which is still poorly understood [46–49]. However, there is evidence to suggest that verterbrate Obps can also function to modulate sensory preception [49].

There are several well-characterized examples in the animal kingdom of how the same sensory stimulus induces dimorphic innate behavioral responses in males and females. In most cases, this strongly depends on the existence of dimorphic neurons and wiring schemes. For instance, male and female mice respond differently to young pups: female mice exhibit maternal behavior towards pups, while male mice show aggressive/infanticidal reactions. Recently, Scott, et al. demonstrated that these dimorphic responses rely on a set of sexually dimorphic dopaminergic neurons within the anteroventral periventricular nucleus (AVPV), the activation of which induces maternal care in female mice, and aggression in male mice [50]. Another study documented differences in sensory processing of pheromone stimuli only in neurons of the medial amygdala, but not in olfactory bulb neurons, suggesting that the dimorphic responses in mice are not encoded at the level of the first and second sensory neurons [51]. A different study demonstrated that eliminating pheromone sensing in adult female mice via surgical removal of the VNO or deletion of the gene TRPC2 produced male-like behavioral responses in females [52]. These findings resemble studies in *Drosophila* in which female flies expressing the male specific Fru^M^ protein display courtship rituals towards other female flies, presumably via developmental feminization of their nervous system [53]. Male flies lacking Fru^M^ do not display courtship towards virgin female flies, but can acquire the potential to court when grouped with other flies [54]. Mice that undergo parasitic infection by *Toxoplasma gondii* present another intriguing example for encoding the valence of the same stimuli differently in males and females. The infection abolishes the innate aversion of female mice to bobcat urine, but does not affect male response to the same stimuli. These behavioral differences are correlated with a sex-specific changes in gene expression in the frontal cortex of male and female mice, including differential expression of olfaction related genes, suggesting that the parasite affects the processing of olfactory information [55].

The above mentioned studies exemplify the central role of dimorphic neuronal circuits in determining sex-specific behavioral responses, and raise the question of how the brain integrates past interaction into the modulation of these behavioral responses. Our findings suggest that such integration occurs not only within the brain, but also in the olfactory sensilla, most likely via an indirect interaction between neurons and auxiliary cells, the result of an intricate interplay by the activity of different types of sensory neurons in male and female flies.

## Materials and methods

### Fly lines and culture

Flies were raised at 25°C in a 12-h light/12-h dark cycle in 60% relative humidity and maintained on cornmeal, yeast, molasses, and agar medium. Canton S flies were used as the wild-type strain. All transgenic fly lines were backcrossed at least 5 generations into a white Canton S background. A UAS-*Obp69aRNAi* line was obtained from the Vienna RNAi collection, *Obp69a-Minos-GAL4* and *Obp28a*-GAL4 flies were obtained from Bloomington, *Lush*-GAL4 was a gift from Richard Benton, *Nomp-A* GAL4 line was a gift from Yun Doo Chung, and UAS *UNC84*-*GFP*, UAS *mCD8-GFP*, *Or67d*-GAL4 and *Or65a*-GAL4 flies were obtained from HHMI Janelia Research Campus.

*Obp69a* GAL4 was generated by inserting a GAL4 coding sequence into the *Obp69a* Locus using homologous recombination. *Obp69a* 5’ and 3’ homology regions of 3Kb were amplified by High Fidelity PCR Kit (Hy Labs) from wild type Canton-S genomic DNA using the following primers: 5' TGTACTTAGGAAAATGGA 3', 5' TTTTGCTTCTCCCCAAAAATTGCTA 3' for the 5’HA arm and 5' CGCTAACCAACCTAAATA 3', 5' AATTTGCTCAAGTTCCCCA 3' for the 3’HA arm. The amplified fragments were cloned into pC31B-JMKS4.2 GAL4-KanR donor vector. This vector contains tdTomato marker under the GMR promoter for visualizing positive donor integration into the MiMIC insertion site of Bloomington stock #35109. Integrant lines were isolated to serve as donors of Obp69a GAL4 DNA substrate for homologous recombination [56] using Bloomington mobilization stock #6934 containing heat-shock-inducible FLP recombinase and I-SceI endonuclease. Transgenic GFP-eyed flies were individually balanced to establish stable lines. The UAS Obp69a-GFP fused transgenic line was generated accordingly; Obp69a coding region was amplified by High Fidelity PCR Kit (Hy Labs) from wild type Canton-S c-DNA library using the following primers: 5’ gctAGATCTatggttgcaaggcatttta 3’ and 5’ attCTCGAGcccaagtagcactattatc 3’ (uppercase letters represent Bglll and XhoI restriction enzyme sites, respectively). The amplified fragment was cloned in frame up-stream to the E-GFP sequence in the pJFRC81-10XUAS-IVS-Syn21-GFP-p10 vector (addgene, UAS) and sent for injection into y1 w67 c23; P{CaryP{attP2 and y1 M{vas-int.Dm}ZH-2A w*;P{CaryP}attP40 sites (BestGene Inc., USA). All transformants were picked from individually injected flies.

### Behavioral assays

All behavioral observations were performed at 25°C, 65% relative humidity and at the same time of day (1h after Lights ON) with 3–4 day old flies unless indicated otherwise.

#### Basic set-up of social conditions

Newly eclosed flies were anesthetized and collected into vials with food, where they were housed separately for three days at 25°C in a 12/12 light/dark cycle. During that time flies were exposed to different social conditions: Single housed (One fly in each vial), Group housed (5 same sex flies in each vial), Group mated (5 same sex flies with 5 opposite sex flies in each vial), Single/Group housed with male scents or Single/Group housed with cVA. Following exposure to different housing conditions, experimental flies were lightly anesthetized using CO2 and decapitated using a micro-scalpel at 1–2 hours after Lights ON. Making sure no antennae were damaged, heads were then frozen in dry ice for total-RNA extraction. We chose to use whole heads and not to surgically remove antennae, as this induces additional variability in the RNA measurements.

#### Mesh/Male scents experiments

Mesh experiments were performed by inserting experimental flies into a food vial containing an Eppendorf tube with food in it. The top of the Eppendorf tube was cut and replaced with a plastic mesh. This enables exposure of scents of group housing for the experimental flies without experiencing direct physical contact with other flies. Five male odor donor flies were placed in the Eppendorf tube. Test flies were placed in the vial for 3–4 days at 25°C, 65% humidity. At the end of the experiment, experimental flies were removed for further processing (decapitation for RNA extraction or behavioral analysis).

#### cVA exposure

Exposure to cVA was performed by adding 1μg or 10μg of cVA dissolved in ethanol onto a filter tip. After the ethanol evaporated, tips were placed in fly food vials and experimental flies were inserted into the vial. cVA was replaced every 24 hours for three days. For expression analysis, heads were frozen (as in the above experimental procedure) for RNA extraction and RT-qPCR analysis. For behavioral tests, three-day old female flies were exposed to cVA either 24h or 1h before behavioral experiments. They were then placed into courtship arenas with virgin 4 days old WT males for behavioral courtship analysis.

#### Optogenetic activation

Light induced activation of the red shifted Channel Rhodopsin *UAS-CsChrimson* was achieved by placing fly vials over a red LED covered plate (40 Hz, 650nm, 0.6 lm @20mA). Activation protocol consisted of three 15 min long activation, with resting intervals of 45 min. First activation started at 1h after Lights ON. Flies were then lightly anesthetized and decapitated, heads were frozen 20–25 minutes after final activation ended.

#### Neuronal inhibition using the inward rectifying channel Kir2.1

Male flies expressing the inward rectifying channel Kir2.1 in Or65a or Or67d neurons and genetic controls were subjected to the following social conditions for three days: single housing or group housing (male cohort), and group housing or group housing with male flies (females cohort). Relative Obp69a levels were determined as previously mentioned. Relative fold change between the different condition for each cohort was calculated by comparing single males to group housed males and comparing group housed females to grouped with males. The fold change ratios were converted to a logarithmic scale of base 2 for linearity. For statistical analysis, One-way ANOVA with Tukey post hoc analysis was used.

#### Neuronal activation combined with inhibition of synaptic vesicle release

Flies expressing Cs-Chrimson and UAS-Shibire^ts^ in Or65a or Or67d neurons were subjected to one of three conditions: (1) Three 15 minutes long optogenetic activations spaced by 45 minutes resting intervals (under constant dark) at constant 22°C serve as positive control. (2) Three 15 minutes long sessions at 28°C under constant dark, spaced by 45 minutes at 22°C, also under constant dark. (3) Three 15 minutes long optogenetic activations at 28°C, spaced by 45 minutes resting intervals with no light and under constant 22°C. 20 minutes following last activation, flies were lightly anesthetized with CO2 and decapitated, as described above. Obp69a relative mRNA levels extracted from whole heads were then quantified using RT-qPCR and compared between the three conditions.

#### Behavioral tests

*Courtship analysis*. 3-4-day old female flies that were raised in groups, either naïve, with male scents through a mesh or with cVA, were inserted into round courtship arenas (0.04 cm^3^ in volume) at Lights ON + 1h together with 4-day old WT naïve males. Courtship arenas were placed in behavior chambers, under controlled temperature and humidity (25°C, 70% humidity). Behavior was recorded for one hour from the introduction of male and female pairs using Point-Grey Flea3 cameras (1080×720 pixels at 30 fps). Latency to copulate was quantified for each pair as total time, starting from first wing vibration the male exhibited and ending in successful copulation. Genetic manipulation of Obp69a in female flies was achieved by driving the expression of specific RNAi or Obp69a-GFP using the *Obp69a-Minos-GAL4*, since this enhancer trap does not affect the basal levels of endogenous *Obp69a*.

*Aggression*. 4–7 day old pairs of Single housed male flies were put into round aggression arenas (about 0.08 cm^3^ in volume). A mixture of agarose and apple juice (1% agarose, 50% apple juice) was inserted into arenas to enhance aggressive behavior (described in [57]). Aggression arenas were placed in behavior chambers, as described above. Experiments were performed in similar time of day (Lights ON + 1h). Flies’ behavior was recorded for 30 min with Point-Grey Flea3 (1080×720 pixels at 60 fps). Aggressive behavior was later quantified by counting the number of lunges for each pair, and latency as the time from start of experiment to first lunge for each pair. Genetic manipulation of Obp69a in male flies was achieved by driving the expression of specific RNAi or Obp69a-GFP using the *Obp69a-Minos-GAL4*, since this enhancer trap does not affect the basal levels of endogenous *Obp69a*.

### Molecular methods

Total RNA was extracted from frozen intact fly heads using TRIZOL reagent. Each sample consisting of 15 frozen heads unless otherwise stated. cDNA was synthesized from total RNA extracts using BIORAD cDNA synthesis kit. cDNA samples were used as templates in a RT-qPCR machine (BIORAD CFX96) using primers for *Obp69a*, *lush*, *cyp6a20*, *est-6*, *obp28a*. relative expression was quantified by ΔΔCT method using *rpl32* as a loading control. Each sample was run in triplicates. Each experiment was repeated at least three time using independent sets of genetic crosses.

*Obp69a* Primers:

F–CCTACGATCATAAAGCAGGTGAGA

R–TCACCGACTTGTCAATCACATCT.

Lush primers:

F–CGCAGGATCTTATGTGCTACAC

R–CATTTCCGGGGGAACCAGAT

*Est-6* primers:

F—AGCACGCAGGAGTCATTGGA

R—CGTCACCGTCTACAGTTCCAAAA

*Cyp6a20* primers:

F—TACTGGAAGCGCCGGGGCATTC

R—CCTCATGGTCTCATCAATGACC

*Obp28a* primers:

F—ATGCCTATCTGCAGGAAATG

R—GCGTCCAGAATTCCGATGTT

*RPL32* primers:

F—ATCGATATGCTAAGCTGTCGCA

R—GGCATCAGATACTGTCCCTTGAAG

### Statistical analysis

#### Statistical analysis

For RT-qPCR experiments, the average relative expression of three, or when indicated 4,5 or 6 independent experiments (every sample within each repeat consisted of 15 flies), were analyzed using Two-Sample T test (Student’s T test) for two groups, or by using One-Way ANOVA with Tukey post-hoc analysis for 3–4 groups. Behavioral experiments were analyzed using One-Way ANOVA with Tukey post-hoc analysis for the groups of experimental flies compared with two genetic controls. When comparing only two groups (Fig 5D), Two-Sample T-test (Student’s T test) was used. All behavioral data were tested for normality. Bonferroni correction for multiple hypothesis testing was applied for each set of related tests which aimed to investigate the same goal of research (indicated in figure legends).

### Confocal imaging

Fluorescent images were captured using Leica SP8 confocal microscope.

## Supporting information

S1 Fig Social conditions, male scents and cVA do not affect est-6 and Lush expression. Relative est-6 (A,B) and Lush (C,D) expression levels in males (A,C) and females (B,D) were quantitated by RT-qPCR under different social conditions, or when exposed to male scents or cVA for three days. Statistical significance was determined by one way ANOVA, Error bars signify SEM n = 3 independent experiments with 10–15 fly heads/sample. P>0.05 for all cases.
(TIF)

S2 Fig Artificially changing Obp69a levels in females does not have a behavioral effect without prior exposure to male scents. **A**. Down regulation of Obp69a using RNAi does not affect receptivity of virgin females that were not exposed to male flies/scents or cVA. Statistical significance was determined by One-way ANOVA, P>0.05, n = 18. **B.** Over-expression of Obp69a using *UAS-Obp69a-GFP* does not affect receptivity of virgin females that were not exposed to male flies/scents or cVA. Statistical significance was determined by One-way ANOVA, P>0.05, n = 18.
(TIF)

S3 Fig Activation of cVA sensing neurons in grouped housed male flies does not affect Obp69a expression level. **A.** Obp69a mRNA levels were analyzed following three consecutive optogenetic activations of Or67d positive neurons in male flies that were housed in a group for three days prior to activation. **B.** Obp69a mRNA levels following three consecutive optogenetic activations of Or65a positive neurons in male flies that were housed in a group for three days prior to activation. Statistical significance was determined by Students T test, Error bars signify SEM, P>0.05, n = 3 independent experiments with 10–15 fly heads/sample.
(TIF)

S1 Table Raw data of the RT-qPCR experiments. (XLSX)
