# Supplementary figures and images for: Odorant binding protein 69a connects social interaction to modulation of social responsiveness in Drosophila
Source: PLoS Genet. 2018 Apr 9;14(4):e1007328. doi: 10.1371/journal.pgen.1007328 (PMC5908198; doi:10.1371/journal.pgen.1007328)

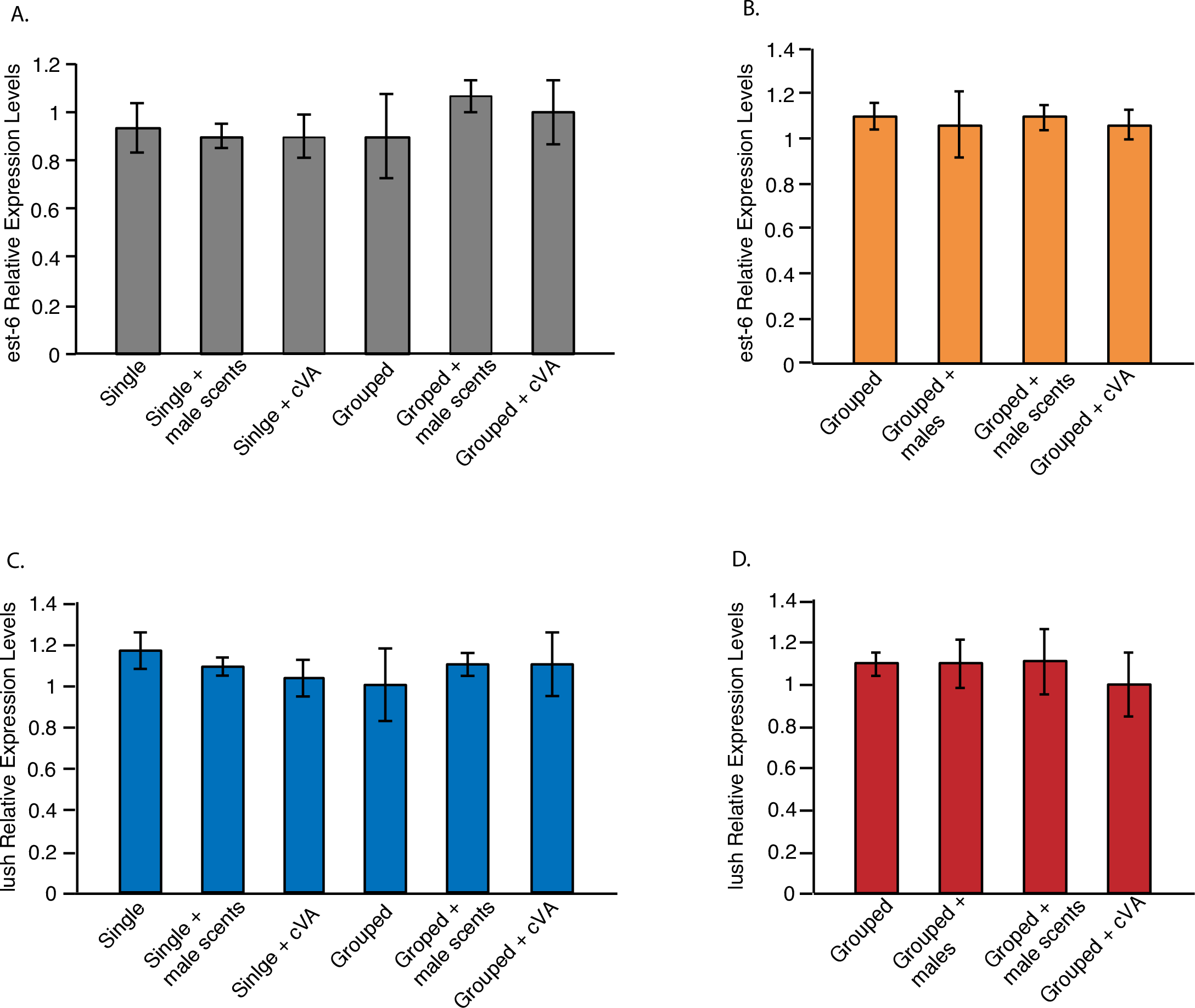

Supplement: S1 Fig — Relative est-6 (A,B) and Lush (C,D) expression levels in males (A,C) and females (B,D) were quantitated by RT-qPCR under different social conditions, or when exposed to male scents or cVA for three days. Statistical significance was determined by one way ANOVA, Error bars signify SEM n = 3 independent experiments with 10–15 fly heads/sample. P>0.05 for all cases. (TIF) [file pgen.1007328.s001.tif]

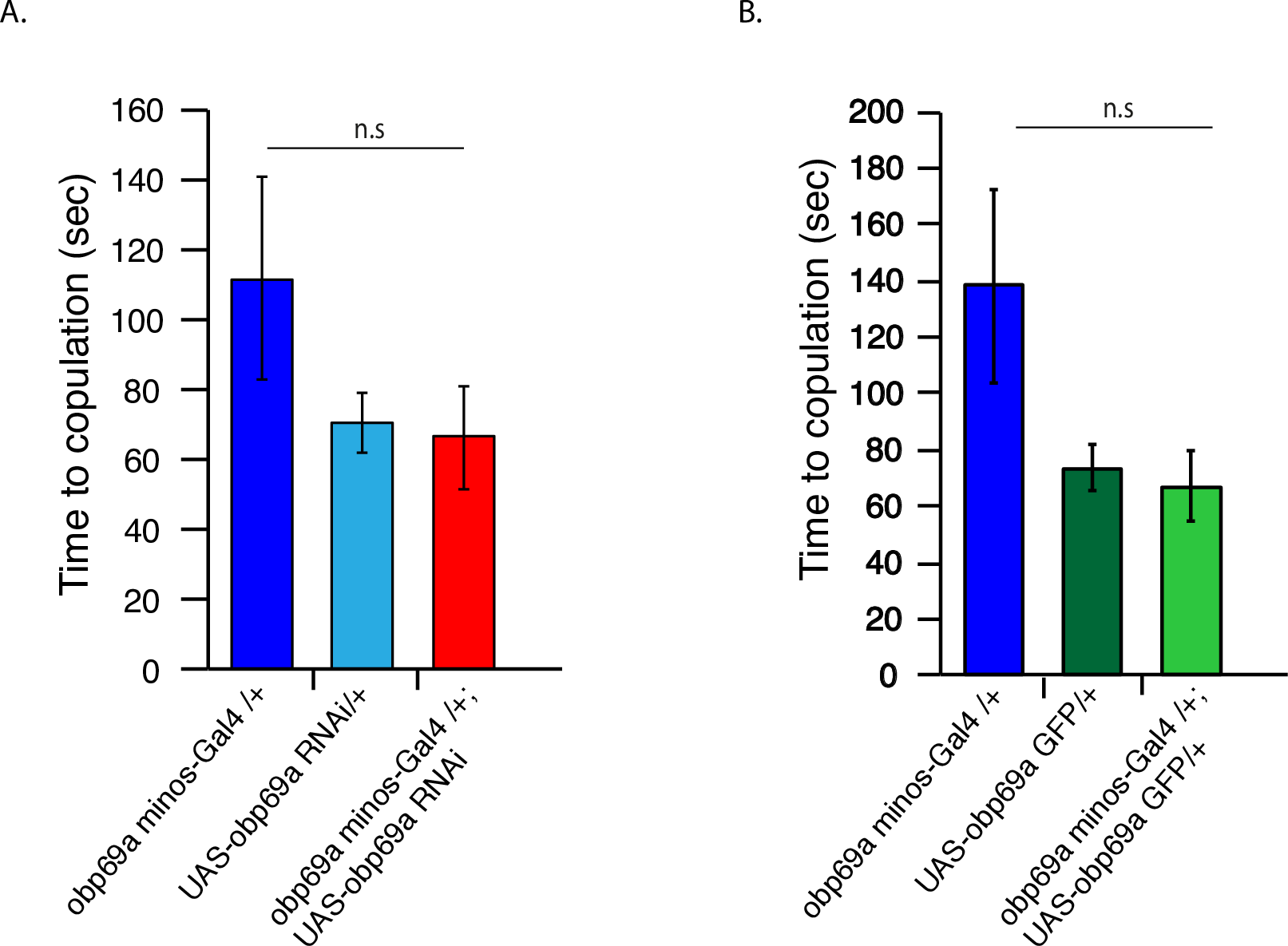

Supplement: S2 Fig — A. Down regulation of Obp69a using RNAi does not affect receptivity of virgin females that were not exposed to male flies/scents or cVA. Statistical significance was determined by One-way ANOVA, P>0.05, n = 18. B. Over-expression of Obp69a using UAS-Obp69a-GFP does not affect receptivity of virgin females that were not exposed to male flies/scents or cVA. Statistical significance was determined by One-way ANOVA, P>0.05, n = 18. (TIF) [file pgen.1007328.s002.tif]

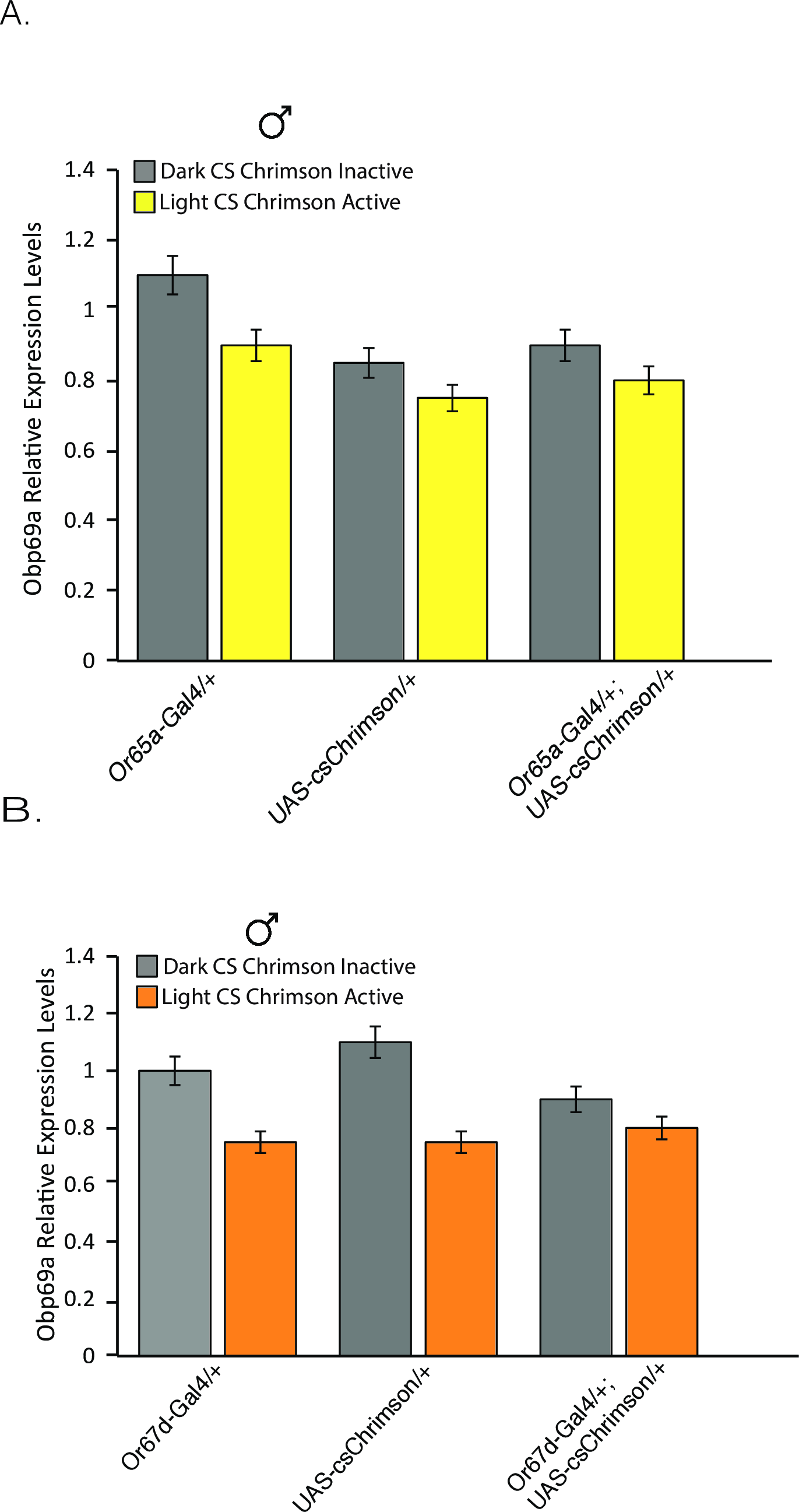

Supplement: S3 Fig — A. Obp69a mRNA levels were analyzed following three consecutive optogenetic activations of Or67d positive neurons in male flies that were housed in a group for three days prior to activation. B. Obp69a mRNA levels following three consecutive optogenetic activations of Or65a positive neurons in male flies that were housed in a group for three days prior to activation. Statistical significance was determined by Students T test, Error bars signify SEM, P>0.05, n = 3 independent experiments with 10–15 fly heads/sample. (TIF) [file pgen.1007328.s003.tif]
